# Selection of internal references for qRT-PCR assays of human hepatocellular carcinoma cell lines

**DOI:** 10.1042/BSR20171281

**Published:** 2017-12-22

**Authors:** Yang Liu, Zhaoyu Qin, Lili Cai, Lili Zou, Jing Zhao, Fan Zhong

**Affiliations:** 1 Department of Oncology, Fudan University Pudong Medical Center, 2800 Gongwei Road, Pudong, Shanghai 201399, China; 2 Department of Gastroenterology, Songjiang Hospital Affiliated First People’s Hospital, Shanghai Jiao Tong University, Shanghai 201600, China; 3 Department of Systems Biology for Medicine, Shanghai Medical College and Institutes of Biomedical Sciences, Fudan University, 138 Yixueyuan Road, Shanghai 200032, China; 4 Translational Neuroscience and Neural Regeneration and Repair Institute/Institute of Cell therapy, The First Hospital of Yichang, Three Gorges University, 8 Daxue Road, Hubei, Yichang 443002, China

**Keywords:** ACTB, HCC, internal references, qRT-PCR, RefFinder, SFRS4, TFG

## Abstract

Selecting internal references is important for normalizing the loading quantity of samples in quantitative reverse-transcription PCR (qRT-PCR). In the present study, a systematic evaluation of reference genes among nine hepatocellular carcinoma (HCC) cell lines was conducted. After screening the microarray assay data of ten HCC cell lines, 19 candidate reference genes were preselected and then evaluated by qRT-PCR, together with *ACTB, GAPDH, HPRT1* and *TUBB*. The expression evenness of these candidate genes was evaluated using RefFinder. The stabilities of the reference genes were further evaluated under different experimental perturbations in Huh-7 and MHCC-97L, and the applicability of the reference genes was assessed by measuring the mRNA expression of *CCND1, CCND3, CDK4* and *CDK6* under sorafenib treatment in Huh-7. Results showed that *TFG* and *SFRS4* are among the most reliable reference genes, and *ACTB* ranks third and acts quite well as a classical choice, whereas *GAPDH, HPRT1* and *TUBB* are not proper reference genes in qRT-PCR assays among the HCC cell lines. *SFRS4, YWHAB, SFRS4* and *CNPY3* are the most stable reference genes of the MHCC-97L under the perturbations of chemotherapy, oxidative stress, starvation and hypoxia respectively, whereas *YWHAB* is the most stable one of Huh-7 under all perturbations. *GAPDH* is recommended as a reference gene under chemotherapy perturbations. *YWHAB* and *UBE2B, TMED2* and *TSFM*, and *GAPDH* and *TSFM* are the two best reference genes under oxidative stress, starvation and hypoxia perturbations respectively. *TSFM* is stable in both cell lines across all the perturbations.

## Background

Hepatocellular carcinoma (HCC) is the most common type of primary liver cancer in adults. It is also the fourth most frequently diagnosed and the third leading cause of deaths among all cancers [1]. The molecular mechanisms underlying the initiation and progression of HCC remain elusive; however, they could most probably result from the changes in the expression levels of several susceptible genes. These cancer-related genes can definitely construct characteristic signal pathways and protein–protein interaction networks, which begin with the occurrence and development of HCC.

It would be helpful to maximize the impact of studies on gene expression. An important component of studying the HCC mechanisms is detecting the expression pattern in the transcriptome scale through high-throughput profiling assays such as microarray and RNA-seq. However, these high-throughput results require further validation in most of the circumstances. Real-time quantitative reverse-transcription PCR (qRT-PCR) has been proven to be a precise and flexible method for measuring a limited number of gene expressions [2–5]. The following two working principles have been used to determine the RNA expression in qRT-PCR: absolute and relative quantifications [6–8]. Absolute quantification primarily relies on the calibration curve for estimating the copy numbers of transcripts [9]. However, estimation bias may be inevitable because of the uncertainty of the initial value assignments. In the majority of cases, researchers determine the difference between concerned samples without considering the absolute abundance of mRNAs and then apply the relative quantification by assessing the fold change between the concerned samples; this process is also termed as the Δ*C*_t_ or Δ*C*_q_ method [10,11].

Several factors need to be considered in the relative quantification, which include the quality and amount of mRNA, the efficiency of the reverse transcriptase, the primer amplification efficiency and the systematic and random variations [12–14]. Proper normalization is an important component of the precise measurement of mRNA and must deal with the cell count or the differences in tissue volume, the RNA concentration and purity variations, the efficiency of the reverse transcriptase and other amplification factors. Although a gene with absolutely stable expression never appears across all samples or treatment regimens, some relatively invariable ones are used as internal references [15–19]. For example, *ACTB, GAPDH, HPRT1* and *TUBB* are frequently used as reference genes in qRT-PCR and Northern blot assay [20–27]. However, the mRNA levels of *GAPDH* are not always constant [28–31] and may contribute to diverse cellular functions [32]. Thus, it is necessary to screen the most stably expressed reference gene(s) for a comparison of each individual expression.

In the present study, the most stably expressed 19 reference candidate genes were preselected from the microarray data of ten HCC cell lines and the stabilities of these putative reference genes together with *ACTB, GAPDH, HPRT1* and *TUBB* were validated by qRT-PCR.

## Methods

### Cell lines and treatments

The following nine HCC cell lines were used in the present study: Huh-7, Hep3B, PLC/PRF/5, MHCC-97L, MHCC-97H, HCCLM3, SNU-398, SNU-449 and SNU-475. All the eight cell lines, except Huh-7, were from hepatitis B virus (HBV)-infected HCC patients. MHCC-97L, MHCC-97H and HCCLM3 were obtained from the Liver Cancer Institute, Fudan University (Shanghai, China) [33]. Huh-7 (catalogue number TCHu182) [34], Hep3B (catalogue number TCHu106) [35] and PLC/PRF/5 (catalogue number TCHu119) [36] were obtained from Shanghai Cellular Institute of Chinese Academy of Sciences (Shanghai, China). These six cell lines were grown in Dulbecco’s modified Eagle’s medium (DMEM; HyClone, U.S.A.) and supplemented with 10% FBS (Biochrom, Germany) and 1% penicillin/streptomycin (HyClone, U.S.A.). SNU-398 (ATCC® number: CRL-2233™), SNU-449 (ATCC® number: CRL-2234™) and SNU-475 (ATCC® number: CRL-2236™) were obtained from the American Type Culture Collection (ATCC) [37] and were cultured in Roswell Park Memorial Institute (medium) (RPMI)-1640 (HyClone, U.S.A.) supplemented with 10% FBS and 1% penicillin streptomycin. All the nine cell lines were maintained at 37°C in a 5% CO_2_ humidified incubator. The cells were grown to 80–90% confluence and harvested three times within ten passages. All the cells were periodically checked to ensure that there is no mycoplasma contamination.

Huh-7 and MHCC-97L cells were respectively treated with cisplatin and sorafenib (Selleck, U.S.A.) dissolved in DMSO for at least 24 h. The final concentrations of cisplatin and sorafenib were 7 and 5 µmol/l in Huh-7 cells respectively, while the final concentration of both cisplatin and sorafenib was 10 µmol/l in MHCC-97L cells. Huh-7 and MHCC-97L cells were treated with H_2_O_2_, with the respective final concentrations being 100 and 2 mmol/l. The starvation of Huh-7 and MHCC-97L cells corresponded with that of the cell lines cultured in 1.5 g/l glucose medium and compared with the control cells grown in 4.5 g/l high-glucose DMEM. Hypoxia was stimulated in the cell lines cultured in 2% O_2_ incubator for at least 24 h. The cell-counting kit-8 (CCK-8) cell proliferation assays (Dojindo, Japan) of Huh-7 and MHCC-97L cells were performed under the hypoxia stimulations (Supplementary Figure S3). Cell cycle analysis of the original and 5 µmol/l sorafenib-treated Huh-7 cells was conducted by flow cytometry using the Cell Cycle and Apoptosis Analysis Kit (Biyuntian, China) (Supplementary Figure S4).

### Preselection of reference candidate genes from microarray data

A total of 48 gene expression microarray (Affymetrix HG U133 Plus 2.0 Array) datasets of ten HCC cell lines (Supplementary Table S1) were collected. The datasets of MHCC-97L, MHCC-97H, HCCLM3 and HCCLM6 and those of two of Hep3B expression assays were obtained from our recent work [38]. The datasets of the other five cell lines and those of four expression assays of Hep3B were obtained from ArrayExpress [39] and Gene Expression Omnibus (GEO) databases [40,41]. Based on the pipeline of calculating the evenness of expression values across all samples, the candidate reference genes with low variation and high levels of microarray hybridization signal intensity (MAS5.0) were screened [42]. The following cutoffs were used: coefficient of variation (CV) <0.11, mean intensity ${\bar{I}}_{i}\;>1000$ and maximum fold-change (MFC) = Max (*I*_i_)/Min (*I*_i_) <1.4, where *I*_i_ denotes the intensity of the gene expression in the arrays of the i-th samples. Max and Min are the maximum and minimum values respectively. The candidate reference genes were analysed based on both probe-level (probe intensities obtained directly from microarray results) and gene-level (sum of all probe intensities) intensities.

### RNA extraction and cDNA synthesis

A total of 5 × 10^6^ cells were collected from each cell line. Total RNA was extracted using TRIzol reagent (Invitrogen) according to the manufacturer’s instructions. The quality and quantity of RNA were measured using NanoDrop ND-1000 (Thermo Scientific, Wilmington, DE, U.S.A.) through OD260/280 and OD260/230 ratios. A total of 500 ng of total RNA was reversed using a PrimeScript® RT reagent kit through poly-dT and random hexamer primers (TaKaRa DRR037) after treating with RNase-free DNase I (TaKaRa DRR2270). Each sample was replicated three times (biological replicates).

### Design of primers and evaluation of amplification efficiencies

The primers of the 16 genes, namely, *AIMP1, ANP32B, BCL2L13, CNPY3, CUGBP1, ENY2, HNRNPC, RPL22, SEC61B, SFRS4, TFG, TMED2, TROVE2, UBE2D3, UBE2V2* and *YWHAB*, were designed by PerlPrimer v1.1.16. The primers of *TSFM* and *UBE2B* were designed by NCBI primer BLAST. The primers of *UBE2N* and *GAPDH* were designed by Primer Premier 5.0. The published primer set was used for *ACTB* (NM_001101) [29]. The primers of *HPRT1* (164518913c1), *TUBB* (34222261c1), *CCND1* (77628152c1), *CCND3* (209915551c1), *CDK4* (345525417c1) and *CDK6* (223718133c1) were selected from PrimerBank (https://pga.mgh.harvard.edu/primerbank/). To avoid the contamination of genomic DNA, the design of most of the primers was composed of cross exon–intron junctions and boundaries that were as far as possible from each other. Agarose gel electrophoresis and melting curve analysis were performed to assess the expected length of the PCR products. The sequencing of the amplicons confirmed the unique expected products. The amplification efficiencies and the specificity of these primer sets were evaluated using standard curve analysis of five-fold serial dilutions and dissociation curves according to previous descriptions [43,44]. Finally, the most efficient primers were selected (Supplementary Table S3).

### Real-time qRT-PCR

Real-time qRT-PCR was performed using the SYBR® Premix Ex Taq™ Kit (TaKaRa DRR041) according to the manufacturer’s instructions, with minor modifications, in 96-well reaction plates using the Applied Biosystems 7500 Real-Time PCR System. Each sample was prepared in a total volume of 25 µl containing 1 µl of 5 µmol/l primer mix (200 nmol/l of each primer), 12.5 µl SYBR Green master mix, 0.5 µl rhodamine X (ROX) Reference Dye II and 9.5 µl RNase/DNase-free sterile water. The initial denaturation was carried out at 95°C for 10 s, followed by 40 cycles at 95°C for 5 s and 60°C for 34 s. The fluorescence data were collected in the 60°C extension phase, and each cell line was harvested in three biological replicates. Each sample was measured in three technical replicates.

The following five experimental perturbations were used in the present study: cisplatin treatment, sorafenib treatment, H_2_O_2_ treatment, starvation by low glucose and hypoxia. Under each condition, both Huh-7 and MHCC-97L cells were harvested from the three biological replicates, and each sample was measured in three technical replicates. The applicability of the reference genes was assessed by measuring the mRNA expression of *CCND1, CCND3, CDK4* and *CDK6* under sorafenib treatment in Huh-7 cells. The ΔΔ*C*_t_ algorithm was used to calculate the fold changes compared with those in the control samples.

### Evaluation of qRT-PCR results

A web-based tool RefFinder was used to evaluate the stability of the candidate reference genes [45]. RefFinder integrates the geNorm [46], NormFinder [47,48], Δ*C*_t_ [49] and BestKeeper [50] and evaluates the most stable gene. Each algorithm uses slightly different methods that are aimed at estimating both the intra- and the intergroup expression variations and allow the ranking of candidate genes based on the instability score [51]. geNorm operates on the assumption that the expression ratio of two ideal candidate genes is constant. NormFinder can indicate the optimal number of reference genes by calculating the accumulated S.D. Δ*C*_t_ is used to compare the relative expressions of ‘pairs of genes’ by comparing their Δ*C*_t_ values. Therefore, Δ*C*_t_ algorithm can analyse large panels of genes based on the ‘process of elimination’. BestKeeper determines whether the candidate genes are differentially expressed under an applied treatment based on the crossing points. geNorm and NormFinder use the stability (actually instability) value, Δ*C*_t_ uses the average of S.D., BestKeeper uses the S.D. of the crossing points and RefFinder uses the geometric mean of ranking values obtained from the above-mentioned four methods. These indexes are termed as instability scores (the smaller, the better) in the present study.

The result of each candidate gene is dependent on all the other ones because geNorm, NormFinder, Δ*C*_t_ and RefFinder use all candidate genes to compute the instability score, indicating that the same candidate genes with different competitive partners will be scored differently. A candidate gene list with even expression levels is beneficial to assess the global stable centre. Therefore, the iterative method was used to compute the instability score using RefFinder and its submethods, namely, geNorm, NormFinder and Δ*C*_t_, by excluding the most unstable ones in each computing cycle.

### Statistical analysis and visualization

The bubble charts were plotted using Excel. The matrix of the generalized pair plot was generated using the function ggscatmat of the R package GGally [52]. The violin plot was drawn using the web-tool BoxPlotR (http://shiny.chemgrid.org/boxplotr/), which uses the shiny package from RStudio. The reciprocals of the instability scores or the reciprocals of CV of microarray intensities, namely, the stability scores, were used to calculate the Pearson correlation coefficients between the various methods.

### Ethics approval and consent to participate

Experiment materials used in this research were mostly the HCC cell lines. Among these cell lines, Huh-7 [30], Hep3B [31] and PLC/PRF/5 [32] were obtained from Shanghai Cellular Institute of Chinese Academy of Sciences (Shanghai, China). MHCC-97L, MHCC-97H and HCCLM3 were obtained from the Liver Cancer Institute, Fudan University (Shanghai, China) [29]. SNU-398, SNU-449 and SNU-475 were obtained from the ATCC.

### Availability of data and materials

All data generated or analysed during the present study are included in this published article (and its supplementary information files).

## Results

### Preselection

**Figure 1:**
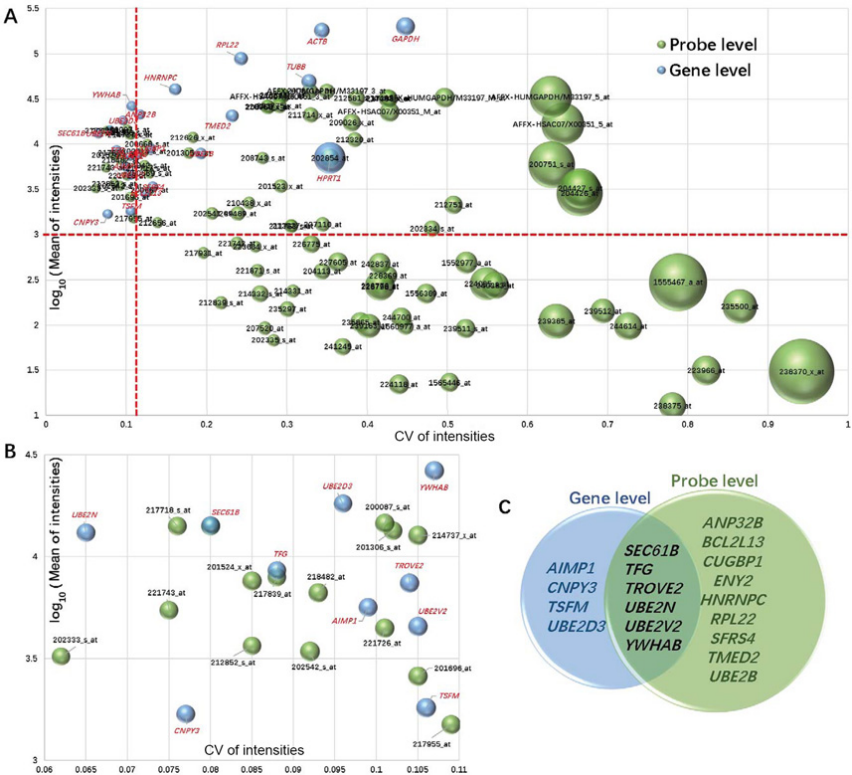
Results of preselecting the reference candidate genes from the expression microarray datasets Items including gene (light-blue bubbles) and probe (light-green bubbles) are denoted by bubble charts of (**A**) all in Supplementary Table S2 and (**B**) pass the preselection cutoff (CV <0.11, ${\bar{I}}_{i}\;>1000$, MFC <0.14). X- and Y-axes are denoted as CV and averaged intensity level respectively. Bubble areas are proportional to MCF values; (**C**) 10, 15 and 6 of the 19 (gene) candidates were preselected from gene, probe and both levels respectively.

Previous studies have always used an arbitrary method of preselection of reference genes to be evaluated [2,12,14,19,47], thereby leading to missing of potential reference genes. Because of the availability of high-throughput transcriptome profiling technologies, such as gene expression microarray and RNA-seq, highly comprehensive candidate gene lists can be obtained from those profiling data [42,53]. In the present study, 19 candidate genes (Figure 1 and Supplementary Table S2) were preselected according to the evenness and the high expression level criteria across all the ten cell line 48 microarray datasets. Moreover, 10, 15 and 6 of the 19 candidate genes were preselected from gene, probe and both levels respectively (Figure 1C). *ACTB, GAPDH, HPRT1* and *TUBB* were added to the candidate gene list for the next step of evaluation.

### qRT-PCR evaluation

**Figure 2:**
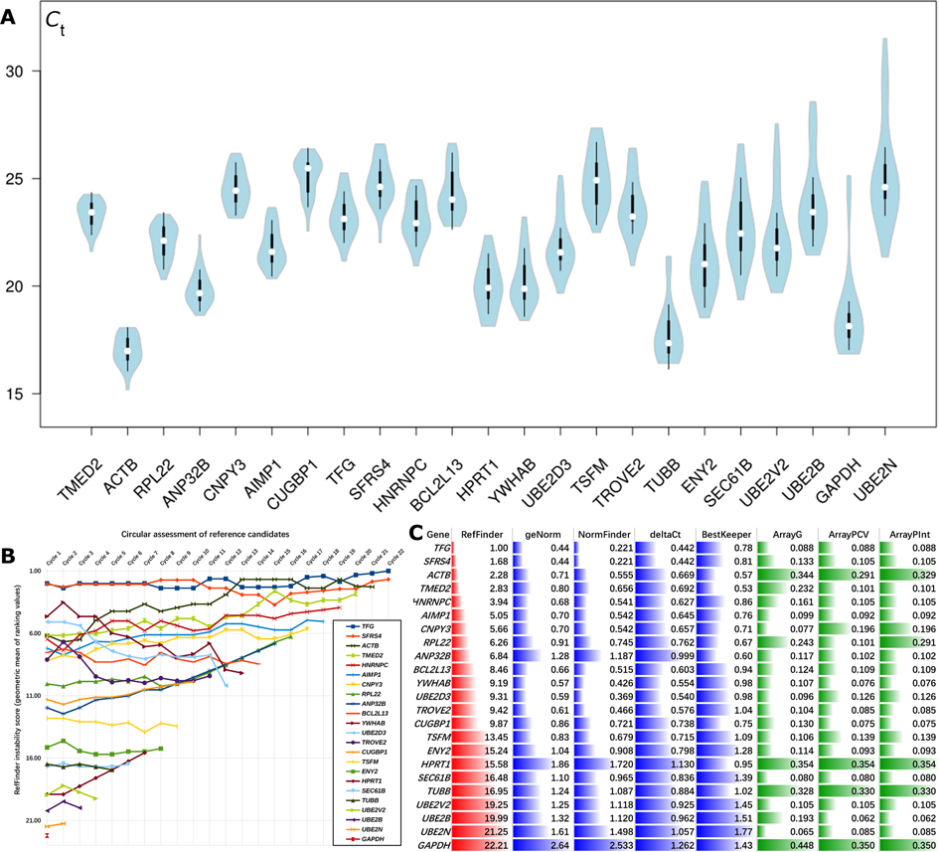
Results of assessing the internal reference genes using qRT-PCR The sample size of each measurement was three biological replicates multiplied by three technical replicates (*n*=9). (**A**) Violin plots of *C*_t_ values of the 23 candidate genes arranged from left to right according to the ascending order of S.D. of *C*_t_. White circles show the medians; box limits indicate the 25th and 75th percentiles; whiskers extend 1.5-times the interquartile range from the 25th and 75th percentiles; polygons represent density estimates of data and extend to extreme values. (**B**) Circular assessment of reference candidate genes by RefFinder. The gene with the highest instability score (geometric mean of ranking values) will be excluded in the next calculating cycle until the final two genes win. (**C**) Final assessments of the internal reference genes by RefFinder and its submethods, such as geNorm, NormFinder, Δ*C*_t_ and BestKeeper, compared with the microarray-preselected results. Microarray-preselected results use the CV of intensities from gene (ArrayG) or probe level. The latter can select a probe with the minimum CV (ArrayPCV) or the maximum intensity (ArrayPInt).

qRT-PCR was carried out to measure the *C*_t_ values of the candidate genes (Figure 2A), and the results showed that *ACTB, TUBB* and *GAPDH* have the highest transcript abundances (lowest *C*_t_ values) and *UBE2N* and *GAPDH* have the largest variations in transcript abundances among the measurements of the nine cell lines with multiple three biological replicates. The RefFinder, which integrates four algorithms, namely, geNorm, NormFinder, Δ*C*_t_ and BestKeeper, was used to evaluate the expression stability from the *C*_t_ values. Through iterative assessment in RefFinder, it was observed that *TFG* and *SFRS4* constantly maintained the top stable positions and finally reached the top two, followed by *ACTB* that finally achieved the third position (Figure 2B and Supplementary Table S4). The individual four submethods revealed slightly different results from those obtained in RefFinder (Figure 2C and Supplementary Table S4). All the three algorithms, except BestKeeper, selected *TFG* and *SFRS4* as the most stably expressed genes in these HCC cell lines, whereas BestKeeper selected *TMED2* and *ACTB* as the top two genes. The iterative ranking results of NormFinder (Supplementary Figure S1A and Table S4) and geNorm (Supplementary Figure S1B and Table S4) were steadier than those of RefFinder. Δ*C*_t_ maintained the instability scores during iterative computations, because a candidate with a high instability score is only determined by all the candidates with low instability scores; thus, the exclusion of the most instable ones does not affect the scores of the others. BestKeeper produces steady results without the need for the iterative strategy. In conclusion, *TFG* and *SFRS4* are the most stable reference genes among these nine HCC cell lines. Regarding the conservative choice, *ACTB* as the third most stable gene performs quite good. All the four algorithms denoted *GAPDH, HPRT1* and *TUBB* as unstable genes in the HCC cell lines.

### Stable reference genes under different experimental perturbations

**Figure 3:**
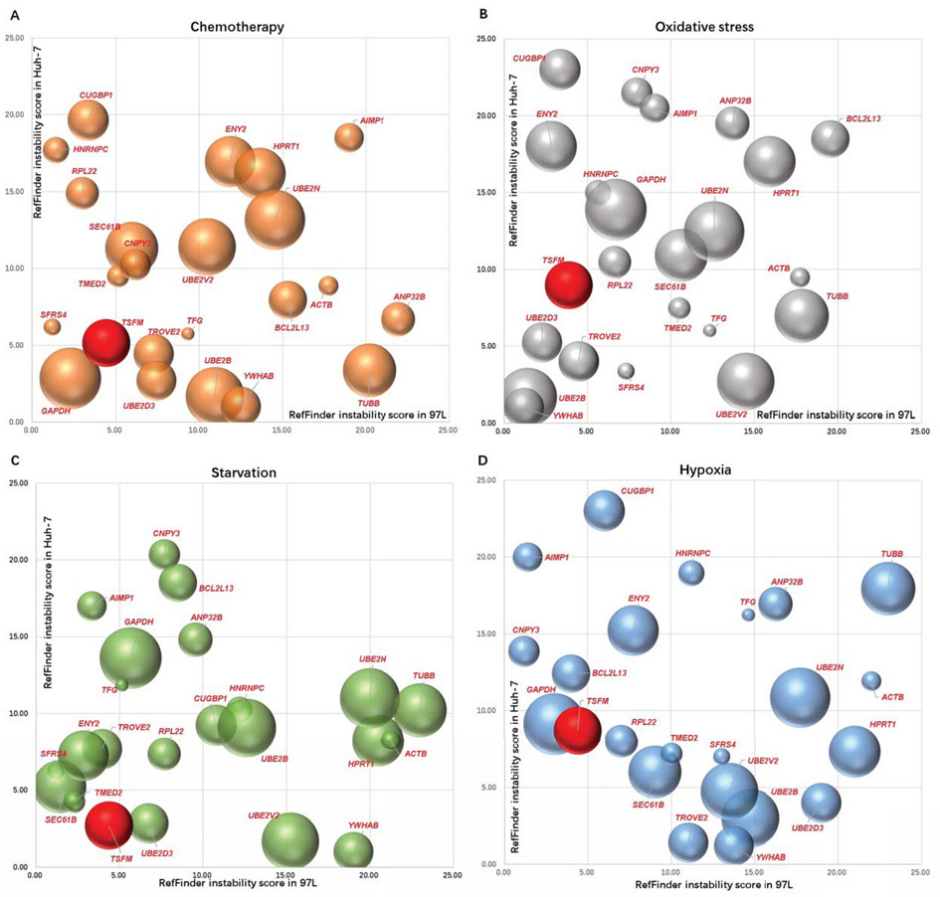
Stability evaluation of reference candidate genes in MHCC-97L and Huh-7 under different experimental perturbations Reference candidate gene performances are denoted by bubble charts under (**A**) chemotherapy perturbations by cisplatin and sorafenib, (**B**) oxidative stress by H_2_O_2_, (**C**) starvation by low glucose and (**D**) hypoxia. The sample size of each measurement was three biological replicates multiplied by three technical replicates (*n*=9). Instability scores from circular assessment of reference candidate genes by RefFinder. The gene with the highest instability score (geometric mean of ranking values) will be excluded in the next calculating cycle until the final two genes win. X- and Y-axes are denoted as ReFinder instability scores from MHCC-97L and Huh-7 respectively. Bubble areas are proportional to ReFinder instability scores from the nine HCC cell lines (Figure 2C). *TSFM*, the best performing reference gene of both cell lines across all the perturbations, is denoted by red bubbles.

The stabilities of the reference genes under experimental perturbations are important. The evaluation of the genes under such experimental perturbations shows complicated patterns. The candidate reference gene behaviour was slightly different in both MHCC-97L and Huh-7 (Figure 3 and Supplementary Table S5). In brief, *SFRS4, YWHAB, SFRS4* and *CNPY3* were the most stable reference genes of MHCC-97L under the perturbations of chemotherapy, oxidative stress, starvation and hypoxia respectively, whereas *YWHAB* was the most stable reference gene of Huh-7 under all the perturbations. From the viewpoint of the perturbations, *GAPDH* can be recommended as a reference gene under chemotherapy perturbations (Figure 3A), while *YWHAB* and *UBE2B* were the two best reference genes under oxidative stress (Figure 3B), *TMED2* and *TSFM* were the two best reference genes under starvation (Figure 3C), *GAPDH* and *TSFM* were the two best reference genes under hypoxia (Figure 3D). *TSFM* showed stable expression in both cell lines across all the perturbations. Moreover, the second echelon should be *TMED2* and *TROVE2*.

**Figure 4:**
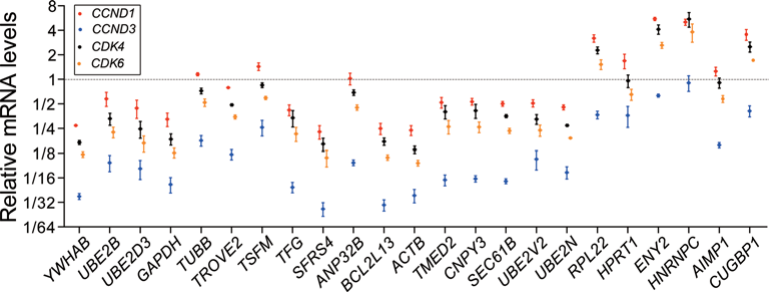
Results of checking the applicability of reference genes under sorafenib treatment in the Huh-7 Dot plot of fold-changes of the four reporter genes under sorafenib treatment that were calculated based on the 23 reference genes. Reference genes arranged from left to right according to the ascending order of performances under chemotherapy perturbations in Huh-7 cells. Circles show the mean values, and error bars show the S.E.M.

To check the applicability of the reference genes under sorafenib treatment in Huh-7 cells, the mRNA expression level changes of *CCND1, CCND3, CDK4* and *CDK6*, which are believed to be down-regulated, were calculated based on the 23 references respectively [54,55]. Most of the 23 candidate genes were suitable for use under the sorafenib perturbation experiments in Huh-7 cells (Figure 4). The Huh-7 cell viability curve declaimed that the proliferation of cells treated with sorafenib was decreased compared with that in the control (Supplementary Figure S3). The cell cycle analysis declaimed that Huh-7 cells were arrested in G_1_ phase under sorafenib treatment compared with that in the control (*P*<0.01, Supplementary Figure S4).

## Discussion

A suitable reference gene should have following two characteristics: (i) it should not have a tissue specific to a gene expression, which is a valid reference gene, and should be expressed under almost all biological and experimental conditions, and (ii) it should have a low CV of expression levels [30]. Previous studies have recommended *SFRS4* and *TBP* as the reference genes in HCV-induced HCC or breast carcinomas [29,43]. Among some putative reference genes selected experientially, *HMBS* and *TBP* were verified to be suitable for reference genes in HCC [4,56]. In the present study, *SFRS4* and *TFG* were screened out by large-scale genomic dataset mining and qRT-PCR as the most stable reference genes in the HCC cell lines. TFG is a TRK-fused gene coded protein, which is a conserved regulator of protein secretion and oncogenesis and has been implicated in neuropathies [57,58]. *SFRS4* encodes a member of the arginine-/serine-rich splicing factor family, which functions in mRNA processing. *SFRS4* expressed in patients with alcoholic liver disease is a relatively stable reference gene used in qPCR technique and is not influenced by steatosis, alcoholic hepatitis, significant fibrosis and cirrhosis [59,60].

*ACTB* is a highly conserved protein and is one of the two non-muscular cytoskeletal actins. *GAPDH* is an important glycolytic enzyme and can catalyse the production of 1,3-bisphosphoglycerate from glyceraldehyde 3-phosphate. *TUBB* encodes the β-tubulin protein and acts as a structural component of microtubules [61]. *HPRT1* is a transferase in the purine salvage pathway and catalyses the conversion of hypoxanthine into inosine monophosphate and guanine into guanosine monophosphate [62]. These four genes are generally used as reference genes in qRT-PCR [20–23]. However, they are not always stable in several samples or conditions. A previous study has shown significantly different expressions of *ACTB* between malignant and non-malignant pairs, upon examination of 16 potential reference gene candidates in 17 untreated prostate carcinomas [15]. Moreover, the expression levels of *ACTB* and *GAPDH* were examined in 80 normal and tumour samples from colorectal, breast, prostate, skin and bladder tissues using qRT-PCR, which revealed that these genes were unsuitable as single reference genes [63]. *HPRT1* has been evaluated in HBV-related HCC studies, but the results were found to be inconsistent [64,65]. *TUBB* has been evaluated as a reference gene in qRT-PCR assays among cell lines and pertubations but the results showed that it is not the most suitable reference gene [66,67]. In the present study, the expression of *ACTB* was found to be quite stable, whereas the other three genes exhibited dramatic variations among the HCC cell lines. To achieve a highly reliable measurement of gene expression, the combinatorial use of two or more reference genes (*TFG/SFRS4/ACTB*) is recommended.

No correlations were found between microarray-preselected and qRT-PCR-evaluated results (Supplementary Figure S2). The results indicated that microarray-based quantification is not sufficiently accurate to distinguish subtle differences in expression stability among genes with a similar performance and is only applicable for an approximate preselection. Reference candidate genes were preselected from the microarray data of ten HCC cell lines but were evaluated only in nine cell lines by qRT-PCR because of the unavailability of HCCLM6 in this step. This situation may introduce some variations between the preselected and the evaluated results. geNorm, NormFinder and ΔCt are highly correlated with each other and have higher consistencies than that of RefFinder. BestKeeper has a lower consistency than those of the other three algorithms and RefFinder, and it is the only algorithm that strongly recommended *ACTB*.

When the stable reference genes were screened under different stimulations, the results of the experimental perturbations showed complicated patterns. None of the candidate genes satisfied the requirements of both the evaluated cell lines under all the perturbations. Considering all the cell lines across all the perturbations, *TSFM* was the most balanced reference gene, followed by *TMED2* and *TROVE2*. The *TSFM* gene encodes a mitochondrial translation elongation factor. The encoded protein is an enzyme that catalyses the exchange of guanine nucleotides on the translation elongation factor Tu during the elongation step of mitochondrial protein translation. A mutation in this gene results in severe infantile liver failure [68] and oxidative phosphorylation enzyme deficiency syndrome [69].

Huh-7 has more consistent reference behaviours than those of MHCC-97L. The best reference gene of Huh-7 cells constantly stuck to *YWHAB* across all the perturbations. *YWHAB* encodes a protein that belongs to the 14-3-3 family of proteins, which mediate signal transduction by binding to phosphoserine-containing proteins. *YWHAB* was initially reported as a reference gene in the present study, but another 14-3-3 family member *YWHAZ* has been selected as a suitable reference gene in several cell lines [66].

Most of the 23 candidates are all suitable under the sorafenib perturbation experiments in Huh-7. Although *YWHAB* and other top ranked candidates, such as *UBE2B, UBE2D3* and *GAPDH*, lead to smaller fold changes of *CCND1, CCND3, CDK4* and *CDK6* than those of *SFRS4, BCL2L13* and *ACTB*, we cannot consider the former group (*YWHAB* etc.) to perform worse than the latter group (*SFRS4* etc.). In fact, the expressions of *CCND1, CCND3, CDK4* and *CDK6* decreased after sorafenib treatment, and the accurate fold-changes remained unknown. The exceedingly reduced value of a target gene can be generated from the actual increment of the reference genes.

It should be mentioned that the present study did not utilize a commonly used liver cell line, HepG2. Although HepG2 cell line and its derivate HepG2/C3A have been annotated as ‘hepatocellular carcinoma’ in the ATCC, the HepG2 cells were in fact isolated from liver biopsy specimens of primary hepatoblastoma (HB, originated from immature liver precursor cells) rather than HCC (originated from mature hepatocytes) [70], and are non-tumorigenic [71]. We believe that HepG2 is out of range of HCC cell lines [72] and have excluded it in our HCC studies designedly.

Although the present study was conducted only within the scope of HCC cell lines, the protocol can be easily applied to other cell lines or specimens. Furthermore, the evaluation of internal reference genes in the present study was conducted only among certain HCC cell lines and perturbations. If internal reference genes applicable to a broader range, such as those between HCC and normal liver or under special stimulations, were to be found, more sample types should be included.

## Conclusion

The combinational use of two or more reference genes, such as *TFG/SFRS4/ACTB*, is recommended in qRT-PCR assays of HCC cell lines. *GAPDH, YWHAB/UBE2B, TMED2/TSFM* and *GAPDH/TSFM* are recommended as reference genes under the perturbations of chemotherapy, oxidative stress, starvation and hypoxia respectively.

## Abbreviations

ATCC: American Type Culture Collection
ACTB: actin beta
CCND1: cyclin D1
CCND3: cyclin D3
CDK4: cyclin dependent kinase 4
CDK6: cyclin dependent kinase 6
CNPY3: canopy FGF signaling regulator 3
Cq: quantification cycle
Ct: threshold cycle
CV: coefficient of variation
DMEM: Dulbecco’s modified Eagle’s medium
GAPDH: glyceraldehyde-3-phosphate dehydrogenase
HBV: hepatitis B virus
HCV: hepatitis C virus
HCC: hepatocellular carcinoma
HMBS: hydroxymethylbilane synthase
HPRT1: hypoxanthine phosphoribosyltransferase 1
qRT-PCR: quantitative reverse-transcription PCR
SRSF4: serine and arginine rich splicing factor 4
TBP: TATA-box binding protein
TFG: TRK-fused gene
TMED2: transmembrane P24 trafficking protein 2
TROVE2: TROVE domain family member 2
TSFM: Ts translation elongation factor, mitochondrial
TUBB: tubulin beta class I
UBE2B: ubiquitin conjugating enzyme E2 B
UBE2N: ubiquitin conjugating enzyme E2 N
YWHAB: tyrosine 3-monooxygenase/tryptophan 5-monooxygenase activation protein beta
